# Preparing for Transmission: Gene Regulation in *Plasmodium* Sporozoites

**DOI:** 10.3389/fcimb.2020.618430

**Published:** 2021-01-29

**Authors:** Sylvie Briquet, Carine Marinach, Olivier Silvie, Catherine Vaquero

**Affiliations:** Centre d’Immunologie et des Maladies Infectieuses, INSERM, CNRS, Sorbonne Université, Paris, France

**Keywords:** *Plasmodium*, malaria, sporozoite, gene regulation, quiescence, transcription, translational repression

## Abstract

*Plasmodium* sporozoites are transmitted to mammals by anopheline mosquitoes and first infect the liver, where they transform into replicative exoerythrocytic forms, which subsequently release thousands of merozoites that invade erythrocytes and initiate the malaria disease. In some species, sporozoites can transform into dormant hypnozoites in the liver, which cause malaria relapses upon reactivation. Transmission from the insect vector to a mammalian host is a critical step of the parasite life cycle, and requires tightly regulated gene expression. Sporozoites are formed inside oocysts in the mosquito midgut and become fully infectious after colonization of the insect salivary glands, where they remain quiescent until transmission. Parasite maturation into infectious sporozoites is associated with reprogramming of the sporozoite transcriptome and proteome, which depends on multiple layers of transcriptional and post-transcriptional regulatory mechanisms. An emerging scheme is that gene expression in *Plasmodium* sporozoites is controlled by alternating waves of transcription activity and translational repression, which shape the parasite RNA and protein repertoires for successful transition from the mosquito vector to the mammalian host.

## Introduction

Malaria is caused by protozoan parasites of the *Plasmodium* genus, and remains a major global health problem in endemic countries. *P. falciparum* is the deadliest species infecting humans, causing more than 200 million cases and 400,000 deaths every year, mainly in sub-Saharan Africa (World Health Organization, 2019). The absence of an available efficacious vaccine and the threat of parasite resistance to anti-malarial drugs emphasize the need for novel anti-malarial intervention strategies. To this end, a better understanding of the biology of the parasite is needed.

**Figure 1:**
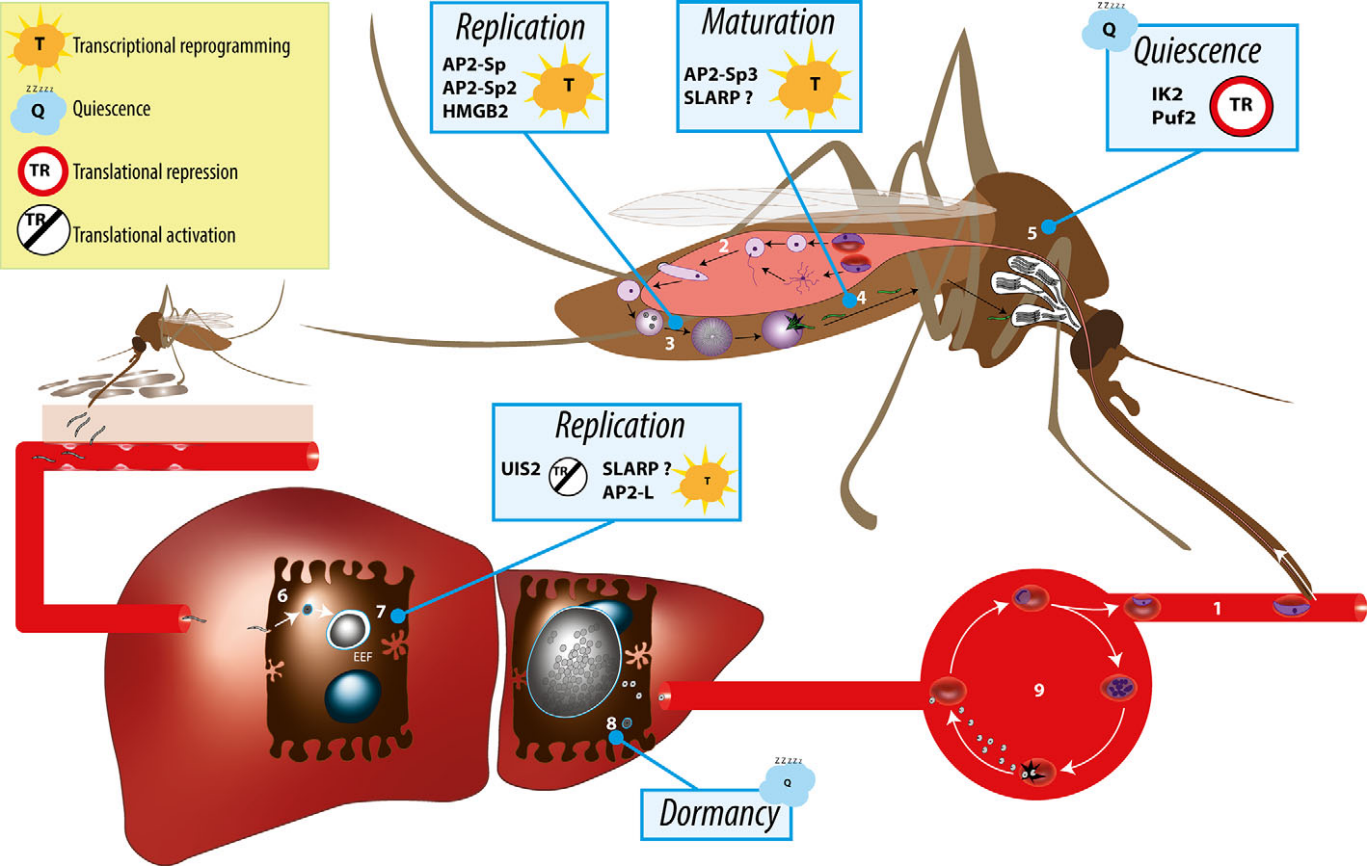
Overview of the *Plasmodium* life cycle and the gene regulation mechanisms controlling parasite transmission from the mosquito vector to the mammalian host. During blood feeding on an infected host, female anopheline mosquito ingest male and female gametocytes (1), which transform into male and female gametes in the insect midgut lumen, followed by fertilization (2) and formation of motile ookinetes that cross the midgut epithelium and transform into extracellular oocysts. Oocysts undergo intense parasite replication (3) until releasing thousands of immature sporozoites that traffic to the salivary glands and mature into infectious sporozoites (4). In the salivary glands, sporozoites persist in a poised state for several days or weeks (5), awaiting transmission, which occurs when the mosquito feeds for blood on a new host. Infectious sporozoites are injected in the host skin and migrate to the liver, where they invade hepatocytes (6) and transform into replicative exoerythrocytic forms (EEFs) (7). In species such as *P. vivax*, some of the parasites do not replicate immediately after invasion but, instead, transform into dormant stages called hypnozoites (8). Parasite replication in the liver culminates with the release of thousands of merozoites into the circulation, which invade erythrocytes and initiate the asexual blood stage cycle (9). Individual factors with functional evidence for a role in transcriptional or post-transcriptional gene regulation during sporozoite development, maturation, or establishment of infection in the liver are indicated.

*Plasmodium* spp. have a complex life cycle that alternates between a mosquito vector and a vertebrate host (**Figure 1**). They are transmitted to mammals by the bite of infected female *Anopheles* mosquitoes, which deposit *Plasmodium* sporozoites in the host dermis (Sinnis and Zavala, 2012). The motile sporozoites actively migrate to the liver, where they invade hepatocytes for an initial and obligatory replication phase. Inside infected hepatocytes, *Plasmodium* resides in a specialized membrane-bound compartment, the parasitophorous vacuole (PV), where sporozoites transform into replicative exoerythrocytic forms (EEFs) (Frischknecht and Matuschewski, 2017). EEFs undergo a dramatic parasite multiplication, lasting 2 to 15 days depending on the parasite species and leading to the release of thousands of merozoites, which invade erythrocytes and initiate the pathogenic blood stage cycle. In some species such as *P. vivax*, a fraction of the parasites inside hepatocytes do not replicate immediately after invasion but, instead, transform into dormant stages called hypnozoites (Adams and Mueller, 2017). Activation of hypnozoites weeks or months after the infectious mosquito bite results in malaria relapses. During blood stage replication, a fraction of the parasites differentiates into sexual stage gametocytes, which upon ingestion by a blood-feeding anopheline mosquito can initiate sporogony in the insect.

Sporozoites develop within oocysts underneath the basal lamina of the mosquito midgut. Upon egress, they are released into the hemolymph and invade the insect salivary glands, where they persist in a poised state for several days or weeks, awaiting transmission, which occurs when the mosquito feeds for blood on a new host. The mosquito-mammal transition is a major bottleneck in the life of the parasite, as only a minor proportion (<20%) of sporozoites successfully invade the salivary glands (Hillyer et al., 2007), from which only a few dozen are inoculated during a mosquito bite (Frischknecht et al., 2004). Sporozoites released from oocysts are weakly infectious for the mammalian host, and gradually acquire infectivity while trafficking in the hemolymph, to become highly infectious after colonization of the insect salivary glands (Touray et al., 1992; Sato et al., 2014).

Comprehensive transcriptome and proteome surveys have shown that phenotypic maturation from immature oocyst-derived sporozoites in the mosquito midgut to mature infectious sporozoites in the insect salivary glands is associated with global changes in the parasite transcriptomes and proteomes (Matuschewski et al., 2002; Lasonder et al., 2008; Mikolajczak et al., 2008; Lindner et al., 2019). Studies based on RNAseq and LC-MS/MS performed with human-infecting species *P. falciparum* and rodent-infecting parasites *P. yoelii* revealed that around 3,500–4,200 genes (representing 60-70% of the genome) have detectable RNAs in sporozoites, while 1,500–2,000 proteins can be detected by mass spectrometry (about one third of the parasite proteins encoded by the genome) (Lindner et al., 2019). Comparison of the midgut and salivary gland sporozoite populations identified up-regulated in oocyst sporozoites (UOS) and up-regulated in infectious sporozoites (UIS) mRNAs and proteins (Matuschewski et al., 2002; Lasonder et al., 2008; Mikolajczak et al., 2008; Lindner et al., 2019). While these studies showed a correlation between RNA and protein abundance for many essential and conserved gene products, they also revealed extensive temporal dissociation between transcript and protein steady state abundance, with two overlapping and independent programs of translational repression that temporally regulate protein expression during sporozoite maturation and following transmission to the mammalian host (Lindner et al., 2019).

*Plasmodium* alternates between active proliferation, in the mosquito midgut and in mammalian hepatocytes and erythrocytes, and differentiation into transmission stages, the gametocytes and sporozoites (**Figure 1**). During progression through this complex life cycle, the parasite switches transcriptional programs. Like in other eukaryotes, gene expression is regulated at both transcriptional and post-transcriptional levels in *Plasmodium*. While gene regulation has been mostly investigated in the parasite asexual and sexual blood stages, fewer studies have looked into the sporozoite stage. In this review, we summarize the current understanding of the gene regulation mechanisms underpinning *Plasmodium* maturation into highly infectious sporozoites capable of invading the mammalian host hepatocytes, establish parasite reservoirs and transmit the disease.

## Challenges in Studying Gene Regulation in *Plasmodium* Sporozoites

Studies of gene regulation in the malaria parasite have been greatly facilitated by the availability of genome sequence data, as illustrated by the first identification of a specific transcription factor (Myb1) in *P. falciparum* (Boschet et al., 2004; Gissot et al., 2005), rapidly followed by the discovery of the plant-like Apetala-2 (AP2) domain family of DNA-binding proteins (Balaji et al., 2005). However, the divergence of protein sequences compared to other eukaryotes, with nearly 30% of the parasite proteins still lacking an annotated function, has hindered the identification of key regulators. Genome-wide mutagenesis studies revealed that 60% of genes without a functional annotation in *P. berghei* show phenotypic defects during the erythrocytic stages, and 36% during the mosquito or liver stages (Bushell et al., 2017; Stanway et al., 2019), while in *P. falciparum* around 1,000 genes of unknown function were predicted to be essential in blood stages (Zhang et al., 2018). Prediction of domain architecture has been used to improve domain recognition methods in *Plasmodium* genomes, expanding new predicted domains up to 30% in *P. falciparum* (platform Plasmobase) (Bernardes et al., 2017; Briquet et al., 2018). While many transcription or translation associated proteins have been predicted in *Plasmodium* through bioinformatics (Roos et al., 2001; Bischoff and Vaquero, 2010; Bennink and Pradel, 2019), only a few of them have been biologically investigated, and it is plausible that other yet unidentified proteins play key roles in the gene regulation networks that concur to the progression of *Plasmodium* through its developmental stages. In addition, the genome extreme AT richness in some species such as *P. falciparum*, with more than 80% AT especially in intergenic regions, is a major obstacle for the identification of *cis*-regulatory elements.

Gene regulation has been mostly investigated in the context of asexual erythrocytic cycle and sexual conversion of *Plasmodium* parasites. In contrast, studies of the mosquito stages, including the sporozoite, are more scarse. Sporozoites must be isolated from anopheline mosquitoes, through hand dissection of the salivary glands of infected females. As a result, only limited numbers of sporozoites can be obtained (typically 10^4^ to 10^5^ per mosquito), resulting in limited sample amounts for downstream analysis of mRNA and/or proteins, with the additional issue of contamination with mosquito cellular content. Despite these limitations, highly sensitive next generation sequencing and mass spectrometry approaches, combined with methods to purify salivary gland sporozoites (Kennedy et al., 2012), have facilitated the deciphering of transcriptomes, epigenomes and proteomes of *Plasmodium* sporozoites (Lasonder et al., 2008; Lindner et al., 2013b; Gómez-Díaz et al., 2017; Swearingen et al., 2017; Zanghì et al., 2018; Lindner et al., 2019; Muller et al., 2019; Hamada et al., 2020).

Nevertheless, steady state mRNA levels result from a balance between mRNA synthesis and mRNA decay, which remain difficult to address in *Plasmodium* sporozoites. Importantly, detection of transcripts and proteins depends on their concentration and on the sensitivity of the analysis. A given messenger might be present in absence of its cognate protein because of translational repression or because the protein is expressed at low level even though it might be functionally competent. Reciprocally, mRNAs present below the detection limit may still be translated into functionally active proteins. Limited sample size is a major hindrance for proteomic detection of transcription factors, which are typically present at low concentrations in eukaryotic cells, allowing to switch on and off rapidly gene expression. Finally, while most studies have looked at bulk salivary gland sporozoites, single cell RNAseq approaches recently revealed some degree of transcriptome heterogeneity in sporozoite populations within the salivary glands (Howick et al., 2019), which could reflect continuing parasite maturation after invasion of the glands and until transmission. Liver stages, especially early exoerythrocytic forms, are typically present in extremely small numbers in experimental culture systems, and pose additional challenges as they cannot be easily isolated from the infected cell. For this reason, the mechanisms underlying stage conversion between quiescent sporozoites to proliferative liver stages remain poorly understood.

## Transcriptional Regulation in *Plasmodium* Sporozoites

The steady state transcriptome of sporozoites is remarkably stable during residence of the parasite in the mosquito salivary glands (Gomes-Santos et al., 2011). This suggests that mature sporozoites are transcriptionally quiescent, a notion further supported by the observation that sporozoites retain infectivity after exposure to the transcription inhibitor Actinomycin D (Silvie et al., 2014). However, exposure of *P. falciparum* and *P. vivax* sporozoites to conditions that mimic the host environment result in profound transcriptome reprogramming, with up-regulation of genes associated with liver stage development (Siau et al., 2008; Roth et al., 2018), illustrating the plasticity of sporozoite transcriptional control.

**Figure 2:**
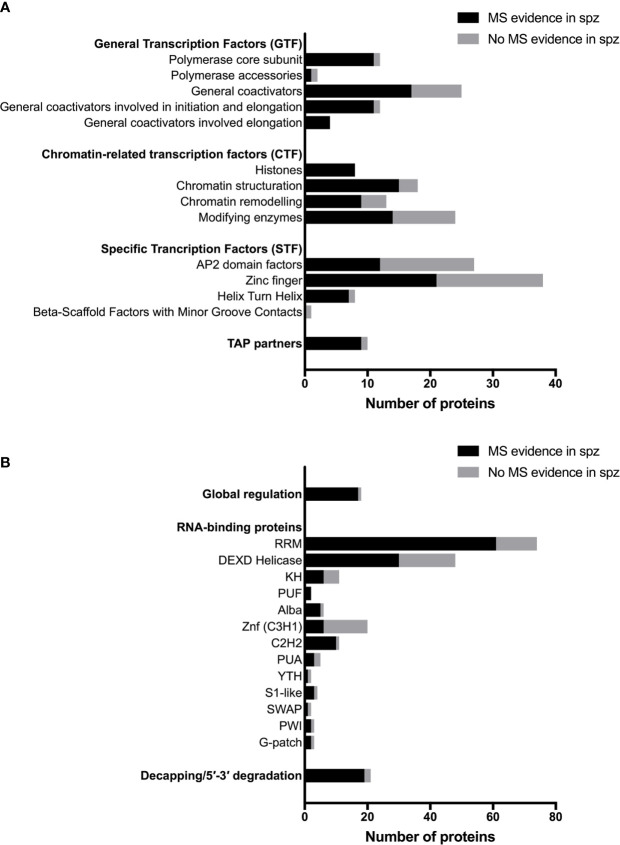
Transcriptional, post-transcriptional and translational regulators in *Plasmodium* sporozoites. **(A)** Number of transcriptional-associated proteins (TAPs), based on the classification of Bischoff and Vaquero (2010). Black bars represent proteins with mass spectrometry evidence in *Plasmodium* spp. sporozoites. Gray bars represent proteins not detected in sporozoites by mass spectrometry. For details on protein identity and mass spectrometry results, see **Table S1**. **(B)** Number of post-transcriptional and translational regulators, based on the classification of Reddy et al. (2015) and Bennink and Pradel (2019). Black bars represent proteins with mass spectrometry evidence in *Plasmodium* spp. sporozoites. Grey bars represent proteins not detected in sporozoites by mass spectrometry. For details on protein identity and mass spectrometry results, see **Table S2**.

*Plasmodium* shares with other eukaryotic organisms common mechanisms for transcriptional gene regulation, including remodeling of the chromatin, post-translational modifications of histones, recruitment of the basal transcription machinery and the concerted action of transcription factors (TFs) that recognize *cis*-regulatory elements in gene promoter regions (Bischoff and Vaquero, 2010; Toenhake and Bártfai, 2019). Many transcription associated proteins (TAPs) have been annotated in *Plasmodium* (Roos et al., 2001; Bischoff and Vaquero, 2010), although few have been biologically investigated in the parasite, especially in sporozoites. They can be classified in three main groups according to their putative function in transcription: general transcription factors (GTF), chromatin-related transcription factors (CTF) and specific transcription factors (STF) (Bischoff and Vaquero, 2010). The three groups are represented in the proteome of sporozoites, albeit at variable levels (**Figure 2A**). **Table S1** lists annotated TAPs and protein detection by mass spectrometry in sporozoites from human and rodent *Plasmodium* species. The lower representation for some of the TAP subcategories could be explained by the absence of expression in sporozoites or by their low abundance in the minute biological material, below the detection limit of mass spectrometry. In eukaryotes, gene expression is largely governed at the level of transcription by stochastic molecular interactions between DNA and TAPs, linked to the fact that most of the proteins exist in cells in very small quantities, which allows switching on and off rapidly gene transcription (Kupiec, 1997). The constant association and dissociation events of DNA with interacting proteins, including histones, contributes to instability, while post-translational modifications of transcriptional regulators can stabilize stochastic gene expression (Kupiec, 1997; Nicolas et al., 2017).

### Chromatin-Associated Regulation in *Plasmodium* Sporozoites

While the regulation of gene expression *via* the binding of specific TFs on their cognate regulatory elements is a central mechanism that controls complex processes of development and differentiation, transcription is for a large part influenced by chromatin organization, in *Plasmodium* as in other eukaryotes (Toenhake and Bártfai, 2019). Chromatin remodeling relies on a variety of readers, writers and erasers, involved in post-translational modifications of histones and driving the highly dynamic structure of nucleosomes underlying gene reprogramming (Tyagi et al., 2016). Architectural remodeling provides plasticity by bringing DNA loops and bound TFs in close proximity to scaffold functional multi-protein complexes. Transcription and genome activity can also be modulated by incorporation of long non-coding RNAs (lncRNA). This well-organized environment, which depends on the number and affinity of regulatory elements, defines transcriptional ecosystems of connected genes that allow the fine tuning of transcription programs for a steady-state level of gene expression (Silveira and Bilodeau, 2018). Disturbance of these ecosystems, including through the action of environmental factors, can lead to reprogramming of gene expression.

Epigenetic regulation is a key control mechanism of *Plasmodium* sexual conversion. In *P. falciparum*, the heterochromatin protein 1 (HP1) plays a central role in epigenetic silencing of the master regulator of gametocytogenesis AP2-G (Brancucci et al., 2014). Derepression of *ap2-g* gene expression depends on gametocyte development 1 (GDV1), itself controlled by an antisense RNA (Filarsky et al., 2018). Furthermore, histone post-translational modifications (hPTMs) act as primary epigenetic regulators of monoallelic expression of variant gene families and phenotypic plasticity in *P. falciparum* erythrocytic stages (Lopez-Rubio et al., 2007; Lopez-Rubio et al., 2009). Recent studies in *P. falciparum* mosquito stages revealed that similar chromatin regulation mechanisms also operate in sporozoites. Analysis by ChIP-Seq of the genome-wide distribution of HP1 and the repressive histone H3 lysine 9 trimethylation (H3K9me3) mark showed that *P. falciparum* (NF54 strain) sporozoites expand heterochromatin at subtelomeric regions to silence blood-stage-specific genes, which correlates with the transcription status of clonally variant gene members (Zanghì et al., 2018). One single *var* gene remained euchromatic, associated with expression of the cognate PfEMP1 protein at the sporozoite surface. Similar results were obtained with *P. falciparum* sporozoites from field isolates, where transcription of a single (but distinct) *var* gene was observed, correlating with the presence of low levels of the repressive H3K9me3 mark in its promoter and with the expression of an antisense lncRNA (Gómez-Díaz et al., 2017). Consistent with observations in *P. falciparum*, the H3K9me3 heterochromatin marks primarily clustered in telomeric and subtelomeric regions in *P. vivax* sporozoites, while marks associated with active transcription, H3 lysine 4 trimethylation (H3K4me3) and H3 lysine 9 acetylation (H3K9ac), were distributed broadly across the chromosomes outside of the telomeric regions, with no overlap with regions under H3K9me3 suppression (Muller et al., 2019).

Sporozoites express several chromatin modifying enzymes, as shown by proteomic analysis, including chromatin writers (SET7 histone methyltransferases, GCN5 histone acetyl transferase) and erasers (HDAC1, Sir2A, Sir2B) (**Figure 2A** and **Table S1**). However, their biological function has not been investigated in sporozoites. One study showed that *P. falciparum* SET7 is distributed throughout the cytoplasm in salivary gland sporozoites, and localizes to distinct cytoplasmic foci adjacent to the nucleus in erythrocytic and liver stage parasites (Chen et al., 2016). Sporozoite proteomes also include chromatin readers, which participate in chromatin structuring and play an important role in transcriptional programming in eukaryotic cells. In *P. falciparum*, the bromodomain protein BDP1 has been reported to bind acetylated histone H3 and plays a role in regulating expression of genes encoding invasion factors in *P. falciparum* blood stages (Josling et al., 2015). BDP1 has been detected in sporozoites by mass spectrometry, as well as the SNF2 helicase and CHD1 chromodomain helicase (**Table S1**). Among TAP partners, the pleckstrin homology domain finger protein PHD1 is expressed in *P. falciparum* and *P. berghei* sporozoites, and could play a role in nodes of connectivity involving epigenetic complexes with bromodomain readers (Hoeijmakers et al., 2019). Again, none of these factors has been biologically investigated so far in sporozoites.

The final step leading to the recruitment of RNA polymerase II and the onset of transcription is chromatin remodeling by increasing nucleosome sliding and accessibility of the chromatin (Agresti and Bianchi, 2003). High-mobility group B (HMGB) proteins are typical members of this category, with architectural properties to recognize distorted DNA and induce stabilization of DNA bending. HMGB proteins appear to be associated with active chromatin (Németh and Längst, 2004; Briquet et al., 2006). HMGB and other chromatin remodeling factors are well represented in the sporozoite proteome (**Figure 2A** and **Table S1**). Interestingly, HMGB2 appears to be a critical regulator of parasite transmission to mosquitoes, since disruption of *hmgb2* gene in *P. yoelii* caused a major reduction of oocyst numbers, associated with the down-regulation of a subset of gametocyte transcripts (Gissot et al., 2008). However, *hmgb2*(-) oocysts were still capable of producing viable sporozoites that were infectious to mice. Although mainly nuclear, HMGB2 was also detected in the cytoplasm of *P. falciparum* gametocytes (Briquet et al., 2006), consistent with the dual life of the protein both as a nuclear protein and as a secreted alarmin (Briquet et al., 2015).

### Sequence-Specific Transcription Factors

Apicomplexan AP2 (ApiAP2) DNA-binding domain proteins form the largest known family of specific TFs in *Plasmodium*, with 27 members in *P. falciparum* (Balaji et al., 2005; Bischoff and Vaquero, 2010). This family includes sequence-specific TFs with homology to the plant apetala 2/ethylene response TF, and are characterized by the presence of one to three DNA-binding AP2 domains (Balaji et al., 2005; Painter et al., 2011). *Plasmodium* AP2 TFs are considered as the main specific TFs responsible for parasite progression through distinct developmental stages (Painter et al., 2011). Twelve AP2 factors have been detected by mass spectrometry in sporozoites from at least one species (**Figure 2A** and **Table S1**), including six that have been detected in all tested species. Reverse genetics studies in *P. berghei* and *P. yoelii* have identified several AP2 factors that play an important role for parasite development in mosquitoes. Four are essential for the formation of infectious ookinetes (AP2-O, AP2-O2, AP2-O3, and AP2-O4), and three are required for sporogony (AP2-Sp, AP2-Sp2, AP2-Sp3) (Yuda et al., 2009; Yuda et al., 2010; Modrzynska et al., 2017; Zhang et al., 2017). Among these, AP2-Sp (Apetala 2 in sporozoites, annotated as AP2-Exp in *P. falciparum*) was one of the first AP2 TFs to be functionally characterized *in vivo* in *P. berghei* with AP2-O. AP2-Sp is expressed from the late oocyst to the salivary gland sporozoite, where it regulates specific gene expression (Yuda et al., 2010). The single AP2 domain of *P. falciparum* and *P. berghei* AP2-Sp binds to the TGCATGCA motif, found in the promoter region of many sporozoite-specific genes (Campbell et al., 2010; Iwanaga et al., 2010; Westenberger et al., 2010), which acts as a *cis*-acting element specific for the sporozoite stage (Yuda et al., 2010; Klug et al., 2018). The AP2-Sp domain was shown to dimerize upon DNA binding (Lindner et al., 2010). *P. berghei* mutants lacking AP2-Sp form oocysts but fail to produce sporozoites (Yuda et al., 2010). Genes under control of AP2-Sp encode proteins associated with a wide range of functions, including sporozoite formation in the mosquito (CSP) (Ménard et al., 1997), sporozoite motility and invasion of the mosquito salivary glands (TRAP, MAEBL) (Sultan et al., 1997; Kariu et al., 2002), sporozoite cell traversal (SPECT, PLP1) (Ishino et al., 2004; Ishino et al., 2005a), invasion of mammalian host cells (P36, P52) (Ishino et al., 2005b) or liver stage development (UIS3, UIS4) (Mueller et al., 2005a; Mueller et al., 2005b). This strongly suggests that additional factors and possibly complex gene regulatory networks participate in the fine-tuning of gene expression during sporozoite development and maturation. Systematic genetic screens in *P. berghei* and *P. yoelii* identified two additional AP2 factors, AP2-Sp2, and AP2-Sp3, as essential for sporozoite production or maturation in oocysts, respectively (Modrzynska et al., 2017; Zhang et al., 2017). Another AP2 TF of the rodent malaria parasite *P. berghei* expressed in liver stages and designated AP2-L was reported to be necessary for parasite development in hepatocytes (Iwanaga et al., 2012). AP2-L disruption does not affect the ability of sporozoites to migrate through cells and to invade hepatocytes but induce an important delay in patency in mice. The AP2-L phenotype is associated with a decreased level of several transcripts specific to the early liver stages (UIS2, UIS3, UIS4, EXP1, and LISP1) but there was no evidence for a binding DNA motif shared within these co-regulated genes (Iwanaga et al., 2012). Finally, among *P. berghei* essential AP2 factors (Modrzynska et al., 2017), some are highly expressed in the sporozoite stage, including AP2-I, which regulates invasion-related genes in *P. falciparum* merozoites (Santos et al., 2017). In fact, among AP2 factors identified in the proteome of sporozoites, six were also identified in the first *P. falciparum* blood stage nuclear proteome, including AP2-I and AP2-Sp (Oehring et al., 2012), showing some overlap in regulatory components between distinct developmental stages of the parasite. AP2-I and other essential AP2 factors expressed in sporozoites possibly participate in the regulation of sporozoite development and/or maturation. As these AP2 factors are refractory to conventional knockout approaches, conditional genome editing will be required for functional characterization in mosquito stages.

Proteomic studies identified other putative specific TFs in sporozoites, i.e., zinc finger and helix turn helix (HTH) proteins (**Figure 2A** and **Table S1**), but their biological role remains unknown. This group includes HMGB3, the only HMGB factor which exhibits one Myb-binding domain (Plasmobase, Vaquero & Briquet personal communication). HMGB3 was detected in the proteome of all tested sporozoite species, as well as the HTH factor ADA2 (Alteration/Deficiency in Activation 2). The first specific TF identified in *Plasmodium*, Myb1 (Boschet et al., 2004; Gissot et al., 2005), is expressed in oocysts and in liver stages, at least at the mRNA level, but not in sporozoites (Howick et al., 2019). A Myb-like TF was recently identified as a master regulator of *Toxoplasma* differentiation, representing a counterpoint of the ApiAP2 factors that dominate the common view of *Plasmodium* gene regulation (Waldman et al., 2020).

In summary, *Plasmodium* sporozoites express many factors potentially involved in transcription regulation, very few of which have been functionally characterized. In addition, unconventional factors could also participate in gene regulation at the transcriptional level, as illustrated by SLARP, which is discussed in a specific section of this review, raising the possibility that non-canonical regulators remain to be discovered in the malaria parasite.

## Post-Transcriptional and Translational Control in *Plasmodium* Sporozoites

*Plasmodium* sporozoites can persist and remain infectious within the salivary glands of the mosquito for several days until they are eventually transmitted to a mammalian host. Remarkably, the parasite mRNA repertoire remains stable during protracted sporozoite residency in the salivary glands (Gomes-Santos et al., 2011). Two control mechanisms are known to operate in sporozoites at the post-transcriptional level: global translation inhibition, at the level of the translation initiation complex, and mRNA-specific post-transcriptional silencing, which typically involves *cis*-regulatory RNA elements and mRNA-binding proteins. Other mechanisms could also be involved, such as mRNA decay mediated by ribonuclease complexes which plays a role in antigenic variation in *P. falciparum* (Zhang et al., 2014), but have not been characterized so far in sporozoites.

Translational regulation plays an important role in quiescent *Plasmodium* transmission stages, i.e., sporozoites and gametocytes, allowing rapid adjustment of gene expression and protein synthesis in response to the new environment that the parasite encounters when transiting from the mosquito vector to the mammalian host, and reciprocally (Bennink and Pradel, 2019). The eukaryote translation machinery is conserved in *Plasmodium*, where bioinformatics predict the presence of initiation, elongation and termination proteins (Cui et al., 2015; Bennink and Pradel, 2019). Many of the factors involved in translation regulation are expressed in sporozoites, as shown by mass spectrometry (**Figure 2B** and **Table S2**). Malaria parasites also express distinct rRNA genes at different developmental stages (Waters et al., 1989), with transcription of S-type rRNA genes taking place as the parasite begins to proliferate in the mosquito (Thompson et al., 1999).

The transient developmental arrest of sporozoites inside mosquito salivary glands implies efficient control mechanisms to prevent premature transformation before transmission. Sporozoite latency in the mosquito is controlled, at least in part, by global inhibition of protein synthesis, mediated through the phosphorylation of the translation initiation factor eIF2α by the sporozoite protein kinase IK2 (Zhang et al., 2010), also termed UIS1 (Matuschewski et al., 2002). *P. berghei* lacking IK2 display a partial loss of infectivity associated with premature transformation of sporozoites in the mosquito salivary glands (Zhang et al., 2010). *In situ* hybridization with oligo-d(T) or labelling with antibodies to the polyA-binding protein showed that sporozoites harbor cytoplasmic RNA granules (Zhang et al., 2010). *Ik2*(-) sporozoites lack such granules and show a general increase in protein synthesis (Zhang et al., 2010). Since global translation inhibition *via* eIF2α phosphorylation is a conserved stress response in eukaryotes, the premature transformation of sporozoites in the absence of IK2 possibly reflects an increased susceptibility of the mutant parasites to stress encountered during residence in the mosquito salivary glands.

Phosphorylation of eIF2α is regulated by the essential phosphatase UIS2, which is expressed in sporozoites and blood stages (Zhang et al., 2016). UIS2 conditional knockout *P. berghei* mutants have a defect in liver stage development, associated with increased levels of phosphorylation of eIF2α. UIS2 may promote reactivation of translation once the sporozoite entered the mammalian host, to allow sporozoite transformation and liver stage development. Intriguingly, UIS2 was recently identified as a PVM protein in *P. falciparum* asexual erythrocytic stages, indicating that UIS2 may have alternative roles (Khosh-Naucke et al., 2018).

Comparison of transcriptome and proteome datasets from *P. yoelii* and *P. falciparum* sporozoites revealed that translational repression affects a large subset of genes, including 70%–80% of UIS (Lindner et al., 2019). Transcriptomic and proteomic data from *P. vivax* sporozoites indicate that translational repression occurs in this species as well (Swearingen et al., 2017; Muller et al., 2019). Post-transcriptional silencing plays a crucial role in female gametocytes, where a defined population of mRNAs encoding ookinete proteins such as P25 and P28 are not translated but instead stably stored in quiescent messenger ribonucleoprotein particles (mRNPs) (Mair et al., 2006; Mair et al., 2010). Transcript-specific translational repression also operates in salivary gland sporozoites, where translation of one of the most abundant mRNAs, encoding UIS4, is inhibited (Silvie et al., 2014; Silva et al., 2016; Lindner et al., 2019). Inside hepatocytes, *Plasmodium* develops within a parasitophorous vacuole (PV) and remodels the membrane of its vacuole by inserting early transcribed membrane proteins such as UIS4 (Mueller et al., 2005a). *uis4* transcripts accumulate in cytoplasmic granules and are translationally repressed in sporozoites, a process that depends on *cis*-regulatory elements contained in the open reading frame of *uis4* (Silvie et al., 2014). UIS4 protein synthesis is activated only after host cell invasion, allowing PVM remodeling. Storage of translationally silent RNA probably permits swift stage conversion yet avoids premature expression of liver stage-specific proteins.

Activation of the translational repression machinery is linked with sporozoite maturation, at least in the case of UIS4 (Silvie et al., 2014), but the effectors remain to be characterized. One possible candidate is the RNA-binding protein Puf2 (Pumilio and fem-3 binding factor homology 2). Puf are evolutionary conserved proteins that typically bind to the 3´ UTR of target mRNAs and repress their translation or induce their degradation (Wickens et al., 2002; Quenault et al., 2011). Silva et al. reported that Puf2 binds to UIS4 mRNA in RNA-immunoprecipitation experiments, and observed an up-regulation of UIS4 protein levels in *puf2(-)* sporozoites (Silva et al., 2016). However, Puf2 is probably not the only effector of UIS4 repression, and overexpression of Puf2 in liver stages has no impact on parasite development in the liver (Lindner et al., 2013a), indicating that Puf2 is not sufficient to repress UIS4 protein expression. Puf2 localizes to cytoplasmic granules in *P. berghei* and *P. yoelii* sporozoites (Gomes-Santos et al., 2011; Lindner et al., 2013a; Silva et al., 2016), and could stabilize sporozoite mRNAs directly or indirectly by regulating other factors controlling RNA metabolism. These storage granules disappear after sporozoite invasion of hepatocytes, possibly releasing specifically bound mRNAs for selective and immediate translation in early EEFs (Lindner et al., 2013a). RNAseq documented genome wide transcriptional alterations in *Puf2*(-) as compared to WT sporozoites, indicating that Puf2, beyond specific translational repression, contributes to mRNA homeostasis in sporozoites (Lindner et al., 2013a). *Puf2*(-) sporozoites can invade hepatocytes and differentiate into EEFs, indicating that Puf2 is not required for liver stage development (Müller et al., 2011; Lindner et al., 2013a). However, Puf2 is essential for maintaining sporozoite infectivity during prolonged parasite residence in the mosquito salivary glands (Gomes-Santos et al., 2011; Müller et al., 2011; Lindner et al., 2013a), a function that relies on the protein RNA-binding domain (Lindner et al., 2013a). Over time, *Puf2*(-) sporozoites transform prematurely into round forms resembling EEFs and loose infectivity, a phenotype that is reminiscent of the behavior of *ik2*(-) mutants (Zhang et al., 2010). In fact, *IK2* mRNAs are down-regulated in *puf2*(-) sporozoites, suggesting that their phenotype could be due, to a large extent, to depletion of IK2 (Gomes-Santos et al., 2011; Müller et al., 2011; Lindner et al., 2013a).

Sporozoites express many other putative RNA-binding proteins in addition to Puf proteins (**Figure 2B** and **Table S2**) (Reddy et al., 2015), some of which likely contribute to global or specific control of RNA homeostasis. As an example, ALBA proteins are DNA/RNA binding proteins that have been detected in DOZI/CITH mRNPs in gametocytes (Mair et al., 2010). In *P. yoelii*, ALBA4 has a dual function, first in suppressing the activation of male gametocytes, then allowing semi-synchronous development of sporozoites. ALBA4 localizes in cytoplasmic granules and deletion of *alba4* gene modifies the steady state level of many genes in sporozoites, showing that ALBA4, like Puf2, contributes to mRNA homeostasis (Muñoz et al., 2017).

## SLARP, a Master Regulator of *Plasmodium* Pre-Erythrocytic Development

Once transmitted to the mammalian host, *Plasmodium* sporozoites invade hepatocytes and rapidly transform into round forms, which then grow and undergo multiple nuclear divisions. The Sporozoite and Liver Stage Asparagine-Rich Protein (SLARP, also called SAP1), which is conserved among *Plasmodium* species and specific for the genus, has been identified as a master regulator of liver stage development (Aly et al., 2008; Silvie et al., 2008). Gene deletion of *slarp* in *P. berghei* and *P. yoelii* does not affect parasite blood stage growth, transmission to mosquitoes nor production of salivary gland sporozoites. However, *slarp(-)* sporozoites are non-infective to mice, due to an early developmental arrest in the liver. *slarp(-)* sporozoites invade hepatocytes and transform into small uninuclear EEFs, but fail to replicate and are rapidly eliminated (Aly et al., 2008; Silvie et al., 2008; Manzoni et al., 2014).

SLARP plays an essential role in shaping the steady state mRNA repertoire of sporozoites in the mosquito (Aly et al., 2008; Silvie et al., 2008; Aly et al., 2011). Transcriptome analysis with oligonucleotide microarrays revealed 38 down-regulated and 14 up-regulated genes in *P. yoelii slarp*-deficient sporozoites compared to WT parasites (Aly et al., 2011). Several *uis* genes, including *uis4*, were enriched among down-regulated genes in *P. berghei* and *P. yoelii slarp*(-) sporozoites (Aly et al., 2008; Silvie et al., 2008). The mechanism of SLARP-mediated regulation of gene expression is still unclear. SLARP is a large protein containing long stretches of low complexity, with an overrepresentation of asparagine residues, but lacks any known functional domain such as DNA- or RNA-binding domains. The C-terminal portion of SLARP is highly conserved across *Plasmodium* species and contains two putative nuclear localization signals (Silvie et al., 2008). The cellular localization of SLARP remains controversial, as different studies documented a cytoplasmic distribution in *P. yoelii* sporozoites (Aly et al., 2008; Aly et al., 2011) and a nuclear localization in *P. berghei* sporozoites (Silvie et al., 2008). Based on 3’ and 5’ RACE PCR experiments in *P. yoelii* sporozoites, Aly *et al*. concluded that SLARP depletion causes specific mRNA degradation, suggesting a role at the post-transcriptional level (Aly et al., 2011). However, experiments with reporter centromeric plasmids established that SLARP controls gene expression by acting on the promoter region of target genes (Briquet & Silvie personal communication). Combined with the nuclear localization of the protein in *P. berghei* sporozoites and EEFs (Silvie et al., 2008), we propose that SLARP functions as a master transcriptional regulator in sporozoites and liver stages (**Figure 1**). The absence of nucleic acid binding domain identified in SLARP sequence suggests an indirect role, perhaps in complex with AP2 domain transcription factors or chromatin-associated regulators.

## Hypnozoites

*P. vivax*, unlike *P. falciparum*, is characterized by relapsing infections, caused by the reactivation of dormant hypnozoites in the liver. Relapses account for a major proportion of *P. vivax* malaria cases, and constitute a major obstacle for malaria eradication (White et al., 2014). This phenotype is shared with a few other primate malaria parasites, including the sister species *P. cynomolgi*, which infects Asian macaques (Krotoski et al., 1982a; Krotoski et al., 1982b). The mechanisms underlying hypnozoite formation, persistence and reactivation remain unknown. In contrast with *P. falciparum, in vitro* culturing of *P. vivax* blood stages is not yet feasible (Gunalan et al., 2020), limiting the access to sporozoites since mosquitoes must be infected by feeding on blood collected from individuals carrying circulating gametocytes. In this context, *P. cynomolgi* provides a relevant model to investigate the hypnozoite biology, and recent progress in genetic manipulation of *P. cynomolgi* has opened new perspectives for the study of hypnozoite formation and reactivation (Voorberg-van der Wel et al., 2013; Voorberg-van der Wel et al., 2020).

It has been hypothesized that, in relapsing *Plasmodium* species, cell fate could be predetermined in sporozoites, which may exist in two forms in the salivary glands of infected mosquitoes, tachysporozoites that develop directly into replicating EEFs, and bradysporozoites, which transform into dormant hypnozoites (Lysenko et al., 1977; White et al., 2017). However, transcriptome and proteome analysis of *P. vivax* sporozoites revealed overall similar steady state mRNA and protein compositions as observed in non-relapsing *P. falciparum* and *P. yoelii*, with stage-specific expression of the same genes needed for hepatocyte invasion and liver stage development (Westenberger et al., 2010; Swearingen et al., 2017; Muller et al., 2019). Nevertheless, orthologs with significantly different expression levels between species were observed, as well as highly expressed species-specific *P. vivax* genes with no known ortholog (Westenberger et al., 2010; Swearingen et al., 2017). While the similar transcriptome and proteome profiles of *P. vivax* as compared to non-relapsing parasites does not favor the hypothesis of predetermined sporozoite fate, single cell approaches are needed to determine whether sporozoites form two distinct populations in relapsing species. Also, other mechanisms could differentially operate in sporozoite subpopulations, including chromatin regulation, which could contribute to stochastic generation of phenotypic diversity in sporozoites, as observed in *P. falciparum* blood stages with clonally variant gene expression (Cortés et al., 2012). Again, this hypothesis is difficult to explore without epigenome studies performed at the single cell level. Another possible model is that formation of hypnozoites takes place in the environment of infected hepatocytes, following sporozoite invasion. A phosphatidylinositol 4-kinase (PI4K) inhibitor reduced the number of hypnozoites in a prophylactic regimen but not when administered as a radical cure scheme (Zeeman et al., 2016), supporting the idea that the dormant phenotype is established post-invasion. Cell fate determination may be influenced by environmental factors in the host cell and/or stochastic regulatory events.

A still open question is whether hypnozoites represent a distinct stage or merely correspond to arrested early EEFs. To solve this question, comparison of hypnozoites and early non-arrested EEFs is still needed, yet would require a hypnozoite-specific marker to distinguish and sort dormant and non-dormant parasites. Unfortunately, there is to date no specific marker of hypnozoite identified. Candidate dormancy-related genes have been identified based on *in silico* studies (Carlton et al., 2008; Tachibana et al., 2012; de Souza Ribeiro et al., 2018), but none has been validated yet. The recent identification of LISP2 as an early marker of liver stage development should facilitate the differential analysis of hypnozoite versus developing early EEFs (Gupta et al., 2019). Indeed, transgenic *P. cynomolgi* expressing mCherry under control of *lisp2* promoter allowed for the first time live imaging of hypnozoite reactivation events (Voorberg-van der Wel et al., 2020).

To circumvent the lack of hypnozoite-specific marker, several recent studies have unraveled the transcriptome of *P. vivax* or *P. cynomolgi* cultured hypnozoites in comparison with hepatic schizonts, either after pharmacological enrichment (Gural et al., 2018) or after isolation of hypnozoites and schizonts by laser capture microdissection (Cubi et al., 2017) or sorting of fluorescent *P. cynomologi* by flow cytometry (Voorberg-van der Wel et al., 2013; Voorberg-van der Wel et al., 2017; Bertschi et al., 2018). These studies revealed that hypnozoites express a lower number of genes compared to schizonts, representing a limited number of pathways. Not surprisingly, the level of gene expression was higher in schizonts than in hypnozoites. When comparing transcriptomes across distinct stages, hypnozoites appear to be a transition point between sporozoites and replicating schizonts, supporting the idea that hypnozoites correspond to arrested early EEFs rather than a distinct stage (Muller et al., 2019). These transcriptional studies failed to identify a specific transcriptional marker for hypnozoites, but revealed that despite a general metabolic shutdown hypnozoites continue to express several specific pathways likely involved in the maintenance of dormancy, including ATP homeostasis and chromatin maintenance (Voorberg-van der Wel et al., 2017; Bertschi et al., 2018).

*P. cynomolgi* hypnozoites display the acetylated H4K8 mark of open chromatin, indicating that they are metabolically active with persistent transcriptional activity (Voorberg-van der Wel et al., 2017). One AP2 domain protein (coined AP2-Q) was proposed as a candidate marker of quiescence, together with two other AP2 factors (AP2-O2 and AP2-G2) (Cubi et al., 2017). However, up-regulation of this putative AP2-Q was not confirmed in other studies (Voorberg-van der Wel et al., 2017; Gural et al., 2018). AP2-L, which plays a role during liver stage development (Iwanaga et al., 2012), was equally expressed in hypnozoites and schizonts in both *P. vivax* and *P. cynomolgi*. Another AP2 domain protein (PVP01_0916300) was found to be differentially expressed in *P. vivax* hypnozoites (Gural et al., 2018), and its ortholog was also found to be expressed in *P. cynomolgi* hypnozoites (Voorberg-van der Wel et al., 2017; Bertschi et al., 2018). Although not specific for relapsing malaria parasites, this particular AP2 factor could be involved in the maintenance of dormant liver stage parasites, together with other AP2 factors identified in hypnozoites (Voorberg-van der Wel et al., 2017).

Cubi et al. also noted an up-regulation of the eIF2 kinase IK2 in *P. cynomolgi* hypnozoites, suggesting a possible role of translational repression in liver stage dormancy (Cubi et al., 2017). Analysis of the phosphoproteome of *P. vivax* sporozoites revealed enrichment in DNA and RNA-binding proteins among phosphorylated proteins, raising the possibility that post-translational control may also participate in regulation of dormancy (Swearingen et al., 2017).

Dormancy could be regulated at least in part by chromatin modifications, since inhibitors of histone methyltransferases stimulate the reactivation of *P. cynomolgi* hypnozoites in simian hepatocyte cultures (Dembélé et al., 2014). In agreement with this idea, hypnozoites were found to express genes involved in histone acetylation and methylation as well as chaperone-mediated modulation of nucleosome-histone interactions, in addition to members of the *alba* gene family, which have DNA and RNA binding activities (Bertschi et al., 2018). Finally, non-AP2 transcriptional regulators could also participate in parasite dormancy, including Myb proteins and SLARP. A Myb-like transcription factor was recently identified as a master regulator of bradyzoite differentiation in *Toxoplasma gondii* (Waldman et al., 2020). *Plasmodium* genome encodes Myb-like proteins, including Myb1 (Gissot et al., 2005), for which transcripts have been found in *P. berghei* liver stages in single cell RNAseq experiments (Howick et al., 2019). Intriguingly, SLARP was detected in *P. falciparum*, *P. berghei* and *P. yoelii* sporozoites (Lindner et al., 2013b; Lindner et al., 2019; Hamada et al., 2020), but not in the *P. vivax* sporozoite proteome (Swearingen et al., 2017). Rodent parasites with targeted gene deletion of *slarp* show a complete developmental arrest in early liver stages, prior to replication, resulting in small hypnozoite-like forms in cell cultures (Aly et al., 2008; Silvie et al., 2008; Manzoni et al., 2014). SLARP is conserved across *Plasmodium* species, yet its contribution during liver stage dormancy is still unknown.

## Conclusion and Perspectives

Transmission of *Plasmodium* from the insect vector to a mammalian host is a critical step of the parasite life cycle, and requires coordinated gene expression for development and maturation into infectious sporozoites that can establish infection in the host liver. Although the knowledge of the gene regulatory mechanisms operating in sporozoites is still fragmented, master transcriptional regulators of sporozoite development and maturation have been identified, which probably participate in more complex regulatory circuits that still need to be characterized. In addition, translational repression plays a crucial role in mosquito stages, ensuring that quiescent sporozoites awaiting transmission in the mosquito salivary glands are already prepared for swift transition to the next replicative stage in the liver of the mammalian host. Many questions remain to be addressed, including the role of chromatin regulators, non-coding RNAs and specific TFs in sporozoite development and maturation. Also, the mechanisms underlying sporozoite transformation into dormant liver stages and hypnozoite reactivation in the medically important *P. vivax* still need to be elucidated. Deciphering gene regulation in *Plasmodium* sporozoites not only will help us to better understand development of this unicellular parasite but will also provide insights into many parasite-specific phenomena such as antigenic variation, alternative invasion pathways and dormancy, which may ultimately lead to the development of novel control strategies targeting host-parasite interactions. More importantly, the panel of the parasite regulation machinery distinct from those of its mammalian hosts makes it a promising target for novel antimalarial chemotherapies.

## Author Contributions

SB, OS, and CV drafted the manuscript. CM and SB computed the proteome data. CM drew **Figure 1**. All authors contributed to the article and approved the submitted version.

## Funding

OS is supported by the Laboratoire d’Excellence ParaFrap (ANR-11-LABX-0024), the Agence Nationale de la Recherche (ANR-16-CE15-0004 and ANR-16-CE15-0010), and the Fondation pour la Recherche Médicale (EQU201903007823).

## Conflict of Interest

The authors declare that the research was conducted in the absence of any commercial or financial relationships that could be construed as a potential conflict of interest.
